# Virtual Reality Game for Physical and Emotional Rehabilitation of Landmine Victims

**DOI:** 10.3390/s22155602

**Published:** 2022-07-27

**Authors:** Vera Z. Pérez, Juan C. Yepes, John F. Vargas, Juan C. Franco, Natalia I. Escobar, Leonardo Betancur, Juanita Sánchez, Manuel J. Betancur

**Affiliations:** 1 Facultad de Ingeniería Electrónica, Universidad Pontificia Bolivariana, Medellín 050031, Colombia; juancamilo.yepes@upb.edu.co (J.C.Y.); juan.franco@upb.edu.co (J.C.F.); natalia.escobarr@upb.edu.co (N.I.E.); manuel.betancur@upb.edu.co (M.J.B.); 2 Facultad de Ingeniería en TIC, Universidad Pontificia Bolivariana, Medellín 050031, Colombia; johnfdo.vargas@upb.edu.co (J.F.V.); leonardo.betancur@upb.edu.co (L.B.); 3 Grupo de Investigación Fisioter, Fundación Universitaria María Cano, Medellín 050012, Colombia; juanita.sanchez@fumc.edu.co

**Keywords:** landmines, rehabilitation, therapy, virtual reality game

## Abstract

Landmine victims require an engaging and immersive rehabilitation process to maintain motivation and therapeutic adherence, such as virtual reality games. This paper proposes a virtual reality exercise game called *Exogames*, which works with *Nukawa*, a lower limb rehabilitation robot (LLRR). Together, they constitute the general *Kina* system. The design and development process of *Exogames* is reported, as well as the evaluation of its potential for physical and emotional rehabilitation. In an initial survey designed ad-hoc, 13 health professionals evaluated compliance with various requirements. They agreed that *Exogames* would help the user focus on rehabilitation by providing motivation; 92.3% said that the user will feel safe in the virtual world, 66.7% of them agreed or totally agreed that it presents characteristics that may enhance the physical rehabilitation of lower limbs for amputees, 83.3% stated that it would promote the welfare of landmine victims, and 76.9% responded that the graphical interface and data report are useful for real-time assessment, and would be helpful for four interventional areas in all rehabilitation stages. In a second evaluation, using standardized surveys, five physical therapists and one lower limb amputee tried the *Kina* system as users. They filled out the System Usability Scale (SUS), the Physical Activity Enjoyment Scale (PACES), and the Game Experience Questionnaire (GEQ). The usability of the *Kina* system overall score was 69 (66, 79) out of 100, suggesting an acceptable though improvable usability. The overall PACES score of 110 (108, 112) out of 126 suggests that users enjoyed the game well. Finally, users indicated a positive effect with a good sense of immersion and smooth of gameplay during the tests, as indicated by the GEQ results. In summary, the evaluations showed that *Exogames* has the potential to be used as a virtual reality game for the physical and emotional rehabilitation of landmine victims.

## 1. Introduction

Nowadays, more than one billion people live with some type of disability, and that number is increasing dramatically according to the World Health Organization (WHO). In particular, people with a lower limb disability often experience stigmatization and discrimination when trying to access health care services, and may receive substandard services when health care providers are in remote areas or without adequate transportation options. Even when those patients arrive at the facility, often the stairs at the entrance or the need to attend activities located on floors without elevators pose an accessibility challenge. There are many other examples of how living in today’s society with a disability leads to restrictions in civic, social and domestic life situations.

More than 143,000 people have been victims of landmines and explosive remnants of war (ERW) worldwide. Landmines include antipersonnel landmines (APM), improvised explosive devices (IED), and antivehicle mines (AVM), among others. ERW refers to ordnance left behind after a conflict, including unexploded ordnance (UXO) and abandoned explosive ordnance (AXO). Each year, the number of registered casualties is increasing. In 2020, there were 7073 cases: 2492 deaths and 4561 injuries, which required comprehensive specialized treatment services and assistance. The International Campaign to Ban Landmines suggests that victims’ assistance programs include physical rehabilitation, psychological support, and social inclusion. However, critical challenges remain in the accessibility, quality, or quantity of services [1,2,3]. Physical rehabilitation is an essential treatment for survivors, but many users never complete their rehabilitation process. For a successful rehabilitation, it is important to maintain the user’s motivation [4,5,6,7]. Some experts suggest that outcomes can be more favorable when users emulate desirable exercises. To address these factors, virtual reality rehabilitation (VRR) programs are advocated as a clinical application tool, showing positive results [8]. “Understanding Virtual reality: Interface, application and design” [9] defines virtual reality as a medium composed by interactive computer simulations which measure user’s position and actions and replace or augment an user’s sensory feedback, bringing the sensation of being mentally immerse or present in the simulation (virtual world). With this set of features, VR has proven to be a good tool for rehabilitation, obtaining measurements of the user’s movements and immersing them into a virtual world that increases motivation and enables the execution of tasks similar to daily ones. Lately, VR experiences come with ’head-mounted displays’ (HMD), whose purpose is to occlude reality from the user and present an entirely new virtual world with a stereoscopic display to increase immersion [10]. Studies have shown that VR adds motivation to repetitive exercises because users focus on the scenarios instead of the exercises [5,6,11].

There are different conditions under which human beings release dopamine, including playing video games [12]. Thus, passing a level, obtaining a high score, competing with others, and facing a new challenge can generate pleasurable sensations that contribute to therapeutic adherence and increase the user’s effort to obtain superior results during the therapeutic intervention process.

Minyoung et al. [13] show results above 90% of therapeutic adherence in a group of 25 post-surgical patients with knee injuries who performed rehabilitation through VR and evaluated the pain level through surveys according to the intensity of the exercises.

Putnam et al. [14] reported a successful application of VRR in a study that interviewed 17 therapists who used Nintendo Wii as a gaming platform for brain injury rehabilitation. The study reported that critical factors for the patient–game interface include cognitive and physical abilities, age, and previous gaming expertise. These factors are essential to incorporate into the system design. Additionally, the game should be perceived as fun, meet the therapy goals, and adapt to patients’ needs.

The main contribution of this paper is describing the evaluation of the design and development of *Exogames* exergame, a virtual reality game that uses body movement during actual exercise routines for landmine victims rehabilitation, with three goals:

- To favor the process of physical rehabilitation of lower limbs for amputees;
- To promote the welfare of landmine victims;
- To support capturing and processing real-time data related to exercises execution during therapy.

**Figure 1:**
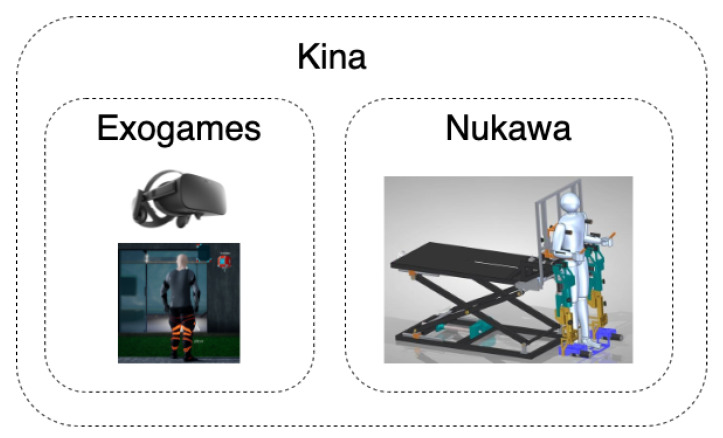
*Kina* system, composed by *Exogames* and *Nukawa*.

**Figure 2:**
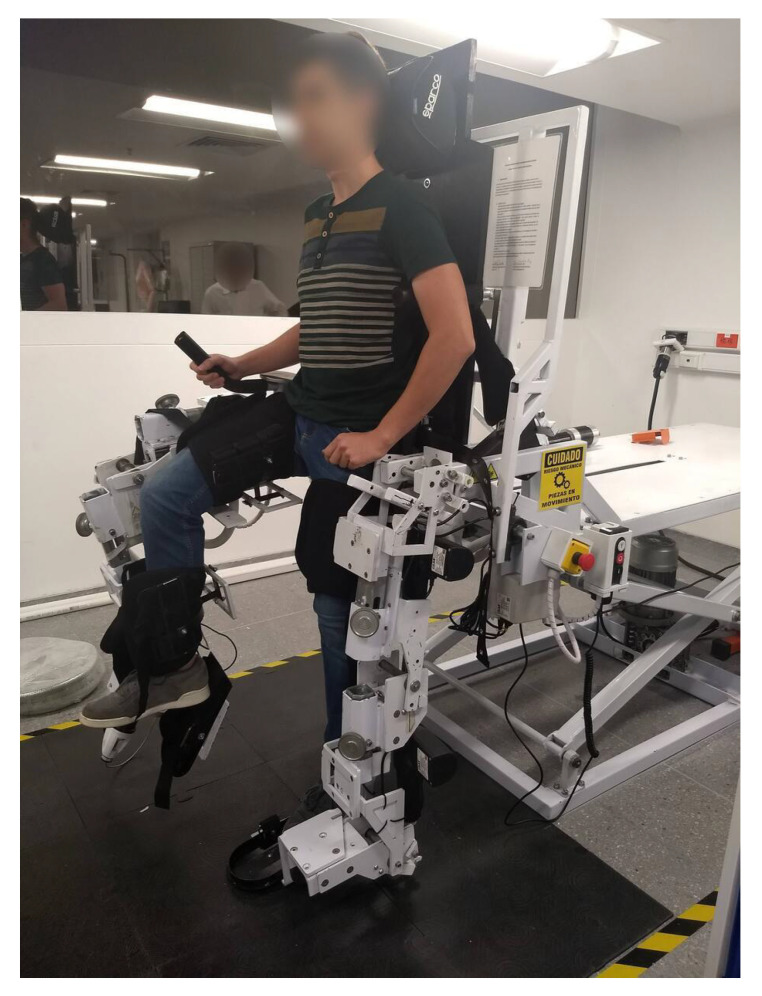
*Nukawa*, the LLRR.

*Exogames* is integrated with the *Nukawa* lower limb rehabilitation robot (LLRR) [15,16,17,18], and together they constitute the overarching *Kina* system, see Figure 1 and Figure 2.

*Nukawa* is the product of technical requirements proposed by an interdisciplinary group formed by physiotherapists, industrial designers, and engineers. The design consists of two limbs, each one composed of a three-link mechanism and an electronic position and force control, i.e., each leg has 3 degrees of freedom (3DOF). The design also has brushless DC motors, power drivers, and position sensors to perform a control strategy capable of generating multiple rehabilitation patterns [15,16,17,18]. When users are strapped into *Nukawa*, position sensors detect the angles of their joints as they interact with the *Exogames* virtual world, and the data are analyzed to generate reports and graphs based on their performance

In order to make an objective evaluation of both *Exogames* and *Nukawa*, different instruments were used to asses usability, enjoyment of physical activity, and gaming experience. The evaluation was done through questionnaires filled out by physical therapists and also by a person who was a victim of an anti-personnel mine (now rehabilitated), including the System Usability Scale (SUS), the Physical Activity Enjoyment Scale (PACES), and the Game Experience Questionnaire (GEQ).

The organization of the paper is as follows: Section 2 presents the methodology considering the criteria for the design and development process of the *Exogames* exergame, and also presents the methods for the exergame assessment by a group of 13 health professionals; subsequently, Section 3 presents results, reporting the description of the developed exergame, the health professionals’ assessment during the first stage of the project, and also the perception of five health professionals and a victim of an anti-personnel mine during the final stage of the project; Section 4 discusses the results and reports the limitations and future work; and, finally, Section 5 presents the conclusions.

## 2. Materials and Methods

This section presents the three phases of the methodology. The first subsection presents the design and development process of *Exogames*, the second one presents the assessment of *Exogames* by health professionals, and the last one presents the *Kina*’s system usability, physical activity experience, and game experience evaluation.

### 2.1. Exergame Design and Development Process

The first step in the design process was a co-creation exercise in which developers and potential users participated. The co-creation exercise objective was to identify a list of requirements for an exergame. These requirements resulted from a brainstorming session of an interdisciplinary group of experts in health, engineering, musculoskeletal rehabilitation, and psychological therapy for victims of violence. The following professionals participated in an initial meeting: a neurologist, a physician conducting a residency in anesthesiology, a psychologist expert in armed conflict, a group of six engineers, a designer, and two physiotherapists.

The moderator conducted a question and answer (Q&A) session and a brainstorming session to identify the game user characteristics and other relevant information for the exergame conception. Table 1 presents the Q&A session questionnaire. Each professional answered the questions they felt comfortable with according to their expertise. They used sticky notes for this purpose. After reading the answers, the professionals had time to discuss and extend their ideas. Afterward, the activity was to join similar ideas and build the first version of the requirement list.

Subsequently, we asked open-ended questions to three lower limb amputees to refine the exergame user characteristics in a semi-structured interview. In addition, it was a pre-validation of the requirements list.

We analyzed the recordings and notes obtained in these sessions, evaluated resource availability from the project perspective, and defined the requirements list. This exercise allowed us to create a user profile, a list of requirements, scenario ideas, and a character description, to develop an exergame according to the target user’s needs. With this input and to complement the co-creation process and exergame development, a company specializing in learning experiences was hired for support during the design and development of *Exogames*. Moreover, a physiotherapist analyzed exercises in physical rehabilitation for landmine victims in pre-prosthetic and post-prosthetic stages and selected seven rehabilitation exercises for the exergame. These sagittal plane exercises are compatible with the capabilities and restrictions of *Nukawa*.

Designers defined a scenario and an architectonic style for the exergame, the main character’s appearance, and its interaction with the virtual world. The result was a Ten-Pager document based on Rogers et al. [19] describing various in-depth aspects of the game such as the title, game story overview, character, gameplay, game world, game experience, and gameplay mechanics. After obtaining the first version of the Ten-Pager, we carried out two feedback and adjustment sessions.

We assembled the virtual reality game in the exergame development process following the Ten-Pager, including 3D models, textures, and sound effects. Next, we used Blender to create the 3D models and Unreal Engine 4 libraries for the rest of the assets. Finally, we used Unreal Engine 4 to import all the assets, create the virtual world, and build the exergame; the last includes the input configuration and mechanics programming to achieve the expected gameplay. The exergame game works for Windows 10 and requires an Oculus Rift headset. The minimum requirements for the computer are a 6th generation Intel Core i5, 8 GB RAM, 5 GB of hard disk space, and an NVIDIA GTX 1060 graphics card.

### 2.2. Exogames Assessment by Health Professionals

After developing the exergame, 13 health professionals were in charge of a first assessment round, 11 of them were physiotherapists and two of them were speech therapists. All had a specialization or Master’s degree, over four years of professional experience, and ages between 25 and 44 years old. First, they received an explanation about the conception of the exergame with the different scenarios, and then they watched a game session. Subsequently, they answered a 22-question survey to assess their perception of the accomplishment of the requirements, the potential for physical rehabilitation and to improve wellness, their perception about the user interface, report, and game usefulness. Table 2 reports questions about the general perception of the *Kina* system. Table 3 depicts questions about intervention areas and graphical interface questions. Table 4 shows questions about the report system and questions about *Exogames*. Finally, Table 5 reports an additional questionnarie about the usefulness of *Exogames*.

### 2.3. Second Assessment Round: System Usability, Physical Activity Experience, and Game Experience

**Figure 3:**
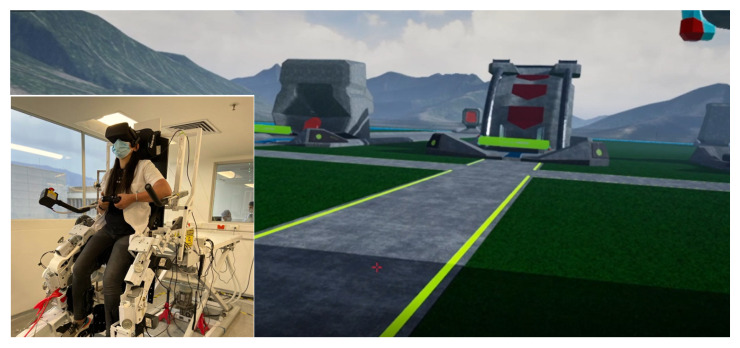
*Kina* user (**left**) and visualization of the exercise machines in the virtual world (**right**).

Five physiotherapists, three women and two men, all of them with a postgraduate, over nine years of professional experience, and ages between 30 and 37 years old, with an average height of 1.68 m and an average weight of 73 kg, evaluated the *Kina* system, that is, the *Nukawa* LLRR in conjunction with *Exogames*. In addition, one male with a lower limb amputee (who had completed his rehabilitation and prosthesis adaptation processes) whose academic level is Baccalaureate, 51 years old, whose height is 1.62 m and whose weight is 75 kg assessed the *Kina* system. None of them participated in the first assessment round, and they all played the exergame before answering the evaluation questions. See Figure 3.

Usability was measured with the SUS, user enjoyment of physical activities was assessed with the PACES, and gaming experience was assessed with the GEQ. All of them signed an informed consent, endorsed by an ethics committee, and then they completed a demographic survey. Subsequently, each of them used the LLRR *Nukawa* to perform several exercises on some of the *Exogames* machines. Finally, they evaluated the usability, physical activity experience, and the game experience. Usability was measured with the SUS, a 10-item scale which measures the usability of a product, where all items are evaluated from 1 “strongly disagree” to 5 “strongly agree” on a 5-point Likert scale. Results may range from 0 for “worst imaginable” to 100 for “best imaginable” [20].

The user enjoyment of physical activities was evaluated using the PACES, since physical activity is required for rehabilitation purposes. We used the 18-item version and all items were evaluated on a 7-point Likert scale. Each participant’s responses were summed and averaged, so the maximum enjoyment score for this scale is 126, which represents the greatest enjoyment while being physically active [21].

The game experience was evaluated using the GEQ, which assesses gaming experience as scores on seven components: immersion, flow, competence, tension, challenge, positive affect, and negative affect with apparently good reliability. Each component is composed by six items, evaluated from 1 “not at all” to 5 “extremely” on a 5-point Likert scale [22,23].

The assessment group also answered open-ended questions about their perceptions of positive aspects, negative aspects, and possible future work in the *Kina* system. The purpose of these questions was to determine elements related to the potential for physical and emotional rehabilitation and characteristics that could favor the system’s applicability.

## 3. Results

This section presents the results of the exergame design and development process along with the assessment results.

### 3.1. Exergame Design and Development Process

Table 6 lists the requirements obtained in the ideation process and the percentage of the 13 health professionals (PHP) who indicated their perception that *Exogames* fulfilled these requirements. Results will be discussed in detail in Section 3.2.

**Figure 4:**
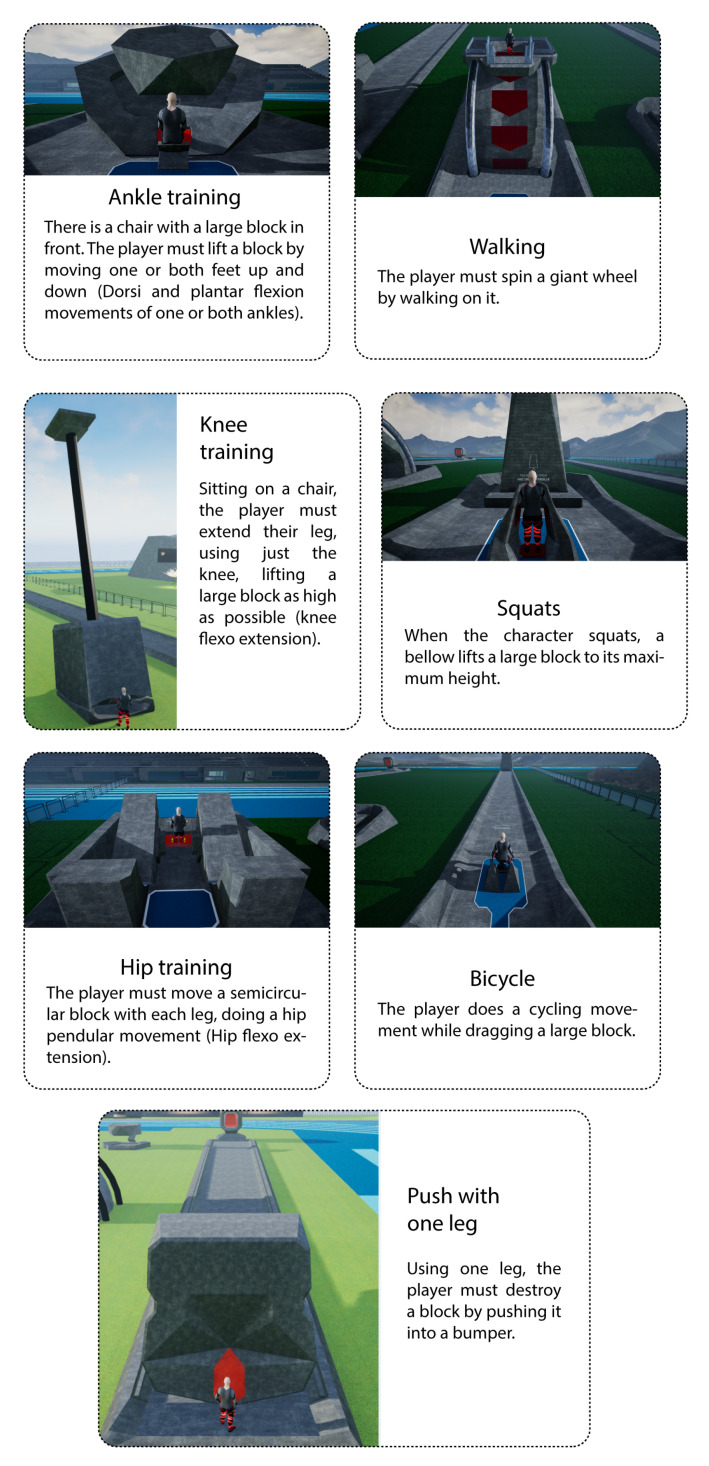
*Exogames* machines and associated exercise movements description.

Seven exercises were selected along with health professionals to implement in *Exogames*. These exercises include movements that start from a neutral position, and the subjects have to perform flexion or extension movements. They may be unilateral, simultaneous, or alternating exercises, as explained in Figure 4, and are executed on the following machines in the virtual world:

- Ankle training;
- Walking;
- Knee training;
- Squats;
- Hip training;
- Bicycle;
- Push with one leg.

Additionally, the health professionals chose the following intervention areas to accommodate the user’s rehabilitation needs and movements:

- Arcs of movement;
- Strength;
- Posture;
- Gait;
- Propioception;
- Equilibrium;
- Pain;
- Sensibility.

#### 3.1.1. User Characteristics and Analysis

The Ten-Pager describes these characteristics for the exergame target user:

- In most cases, potential users may belong to one of two types: (1) People who live in rural areas, perform physical labor, are prone to concrete thinking, have limited access to technology and healthcare, and, in many cases, have been victims of armed conflicts; and (2) Trained military personnel, with better access to technology and healthcare systems.
- People with mobility limitations who feel disabled and dependent due to lower limb amputation. They also feel frustration, trauma, and anxiety, generated by phantom limb pain and the unfamiliarity about the entire rehabilitation process. This lack of understanding may persist throughout the rehabilitation process. They may feel insecure when not using their prostheses since they use them for esthetic reasons rather than functional ones. Their context, including family and close relationships, is a factor that increases or decreases these feelings. The user has felt a change in their self-value, and it feels an obligation to reevaluate their life goals. The satisfaction of meeting challenges is an excellent motivator in their life.
- Occasionally, the person feels phantom limb pain, fear, catastrophizing, hopelessness, or depressive symptoms due to the absence of the limb.
- The potential user wants motivating therapeutic exercises related to everyday activities like playing sports, gym exercises, or dancing. In addition, they find it inspiring to perform activities that are harder to perform after the amputation.
- With time and a rehabilitation process, this person can become very functional, recover their independence, accept the limb’s absence, and decide whether or not to use the prostheses or other braces such as crutches. In the end, the person might not feel incapacitated to return to normal activities and adopt new interests. However, this user may feel nostalgic for not being able to perform specific activities, like dancing or playing some sports.
- The potential user feels motivated to compete against other users in similar rehabilitation conditions. Experiences with people who suffer from a similar or worse condition can inspire others to overcome theirs. It is helpful to keep in touch with other people during rehabilitation.

#### 3.1.2. *Exogames* Overview

*Exogames* is a VR exergame designed to aid in rehabilitation, adaptation, prosthesis acceptance, and users’ progress. It integrates with the *Nukawa* LLRR and the Oculus Rift VR headset for 3D visualization and to perform rehabilitation movements.

When putting on the VR headset, users see themselves as athletes wearing a state-of-the-art exoskeleton in the form of a virtual avatar that they can control by moving their legs in the *Nukawa* system and can reposition themselves in the virtual world using a hand controller.

We designed and developed this exergame to support adherence and enable valuable data collection for rehabilitation therapies. According to the Ten-Pager game story overview, the “International Olympic Committee” announced the integration of a new sport created for cyborgs called *Exogames* for the Paris 2024 Olympic Games. These new athletes have exoskeletons that increase their motor capabilities to participate in the competition. In the exergame, the user’s primary goal is to qualify for the Olympic Games. For this purpose, they should train and then take qualifying tests.

**Figure 5:**
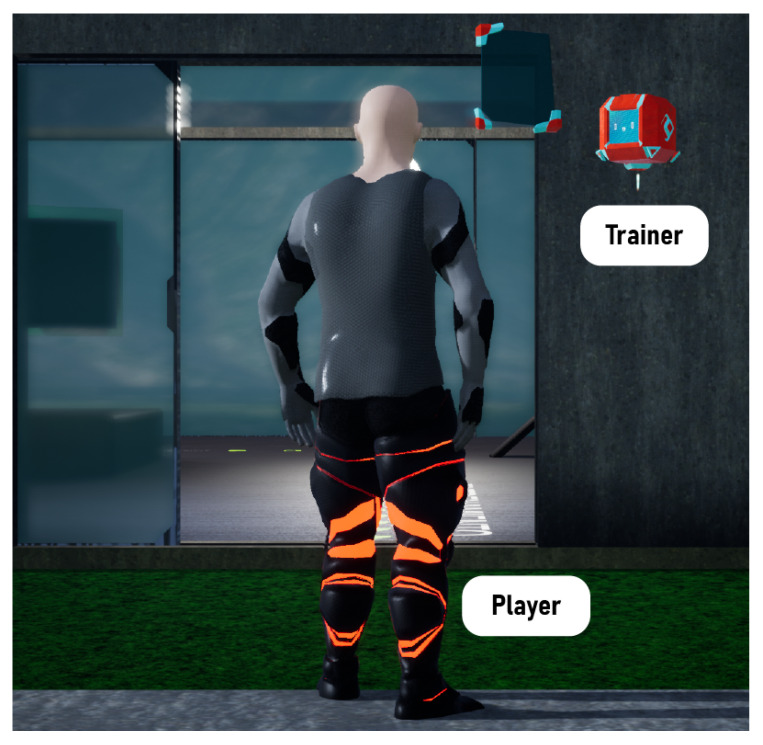
Avatar and trainer in *Exogames*.

The user relies on a virtual trainer that determines an exercise routine and monitors the advances. The trainer is a floating interface on the right side, which communicates the physiotherapist’s recommendations and treatment plan, motivates, gives instructions to perform different activities, and counts repetitions. Figure 5 shows a third-person view of the user avatar wearing the exoskeleton and the trainer, although the game uses a first-person view. A video tour of the virtual world is available at https://youtu.be/L_WysWslZz0 (accessed on 19 June 2022).

**Figure 6:**
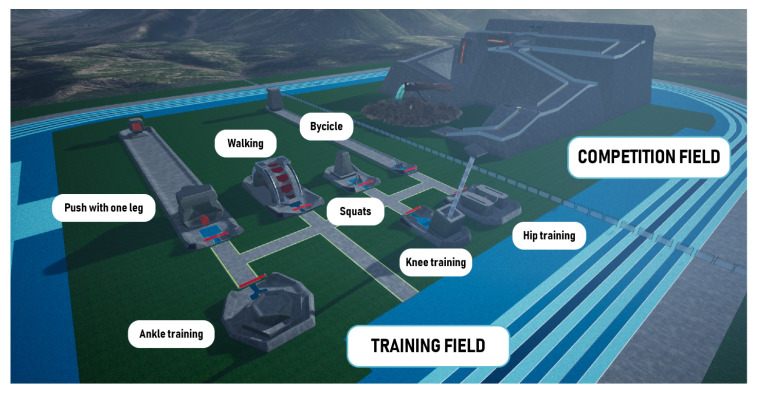
Training and Competition field.

The exergame takes place in a field inspired by the *Atanasio Girardot* sports complex in Medellín, Colombia. The sports activities can be performed in the Training Field or the Competition Field, see Figure 6. In the Training Field, competitors interact with massive exercise machines to perform those exercises. When the physiotherapist determines it is time, according to their progress, users can participate in the challenge at the Competition Field and advance to the next category. Users begin as a rookies, as indicated by the orange lights on the exoskeleton, then evolve to apprentices, wearing yellow lights, and, finally, they earn the green light when ready to participate in the master category of *Exogames*.

The user should pass the qualifying test to advance to the next category of the Olympic Games qualifying process. Then, the physiotherapist may decide the difficulty and intensity of the exercises by increasing the number of series or the number or repetitions in each machine. In this sense, when the user finishes a level, the physiotherapist may configure the difficulty harder so that the player moves to the next category.

The user navigates around the machines in the Training Field to perform the corresponding exercises, one of the seven selected exercises, as shown in Figure 4. The physiotherapist can choose, in each session, the apparatus with which the user will train.

After the training sessions, the user can participate in the ranking competition, once cleared by the physiotherapist. Here, the user must complete challenges in the shortest possible time by using the skills learned during training. The time trial will rank the user in comparison to others. The objective is to create a flow of water, clearing some obstacles, so that it reaches a small flower. Initially, the user heads to the starting line, where the first challenge is to open a gate by walking on a giant stone wheel. Then, the user must kick a large rock that blocks the canal. Next, the user must lift a stone with a bellows, allowing the water to flow to the last challenge. Finally, the user will use a stationary bike to move a tree trunk and aim the water at the flower, to complete the quest.

Every time a user starts a therapy session, they can see their ranking against others. Refer to the Supplementary Materials to watch a video of *Exogames*.

### 3.2. Game Assessment by Health Professionals

**Figure 7:**
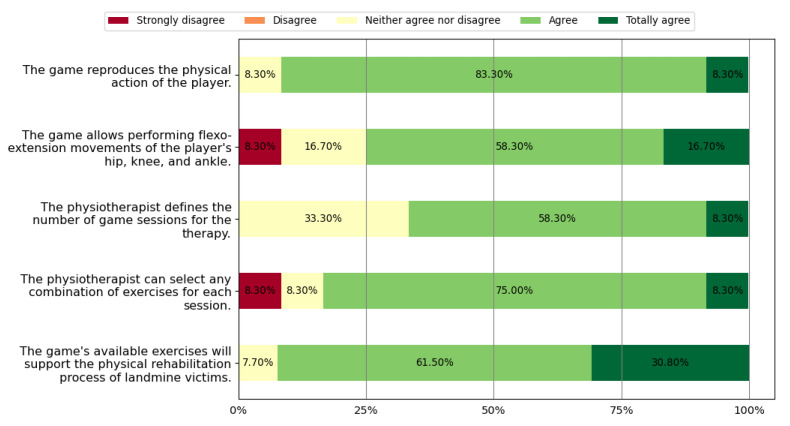
Perceptions related to physical rehabilitation.

**Figure 8:**
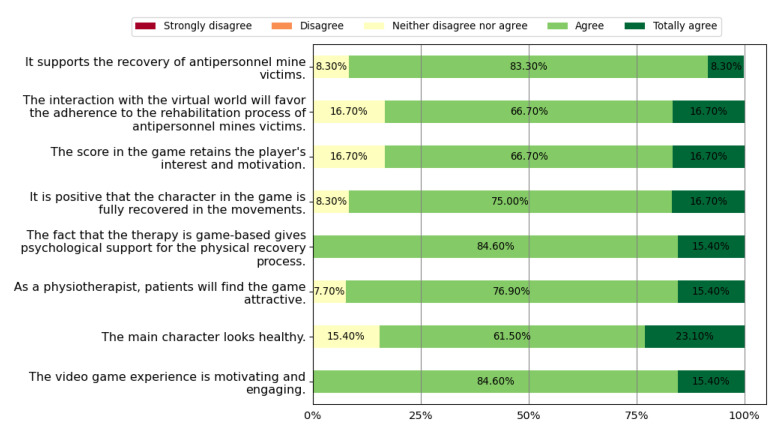
Perceptions related to wellness.

**Figure 9:**
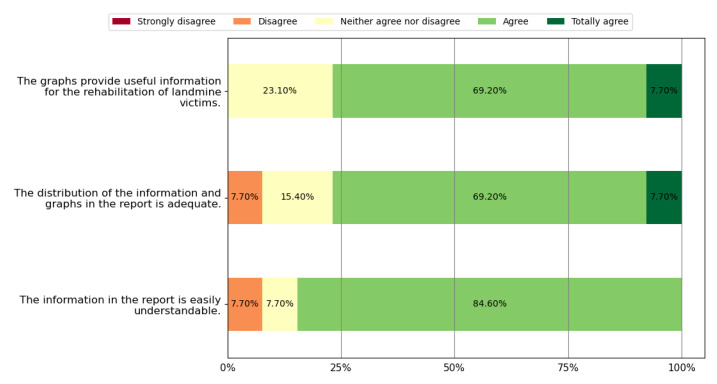
Perceptions about *Kina*’s interface and data reports.

This section presents the results obtained from the survey related to experts’ perceptions according to the methodology reported in Section 2.2. Table 6 shows the PHP who agreed that the game meets the requirements defined at the beginning of the methodology. Figure 7 presents the results of the health professionals’ perception regarding physical rehabilitation potential. In this figure, we can observe that 66.7% of the physiotherapists agreed or totally agreed that *Exogames* meets all five physical rehabilitation requirements. Figure 8 reports the perceptions of health professionals related to wellness. In this figure, we depict that 83.3% of them agreed or totally agreed that *Exogames* meets all eight wellness requirements. In addition, Figure 9 presents the results about the game’s perceived usefulness of its graphic user interface and user rehabilitation status report. In this figure, we evidence that 76.9% of the health professionals agreed or totally agreed that *Exogames* meets all three requirements for the reports and graphic interface. To summarize, 66.7% of all the physiotherapists agreed or totally agreed that *Exogames* meets all the requirements for a VR game for physical and emotional rehabilitation of APM, IED, and UXO victims.

Table 7, Table 8 and Table 9 present the potential benefits that the health professionals reported. In these tables, we can observe that the professionals think that *Exogames* has other benefits related to metrics and visualization, as seen in Table 7. Moreover, they declared that *Exogames* combined with the LLRR would be useful for different physical rehabilitation therapy stages in transtibial amputation, as seen in Table 8. Finally, they stated that *Exogames* could also contribute to therapeutic objectives framed in different areas of intervention, as shown in Table 9.

### 3.3. Kina’s System Usability, Physical Activity Experience, and Game Experience Evaluation Results

*Exogames* obtained the following scores: SUS 69 (66, 79), which means SUS median score 69, with Q1 = 66, and Q3 = 79; for PACES score, *Exogames* obtained 110 (108, 79); with respect to the GEQ score, the results obtained were: flow 4 (3, 4), positive affect 4 (2, 4), immersion 4 (3, 5), competence 4 (4, 5), challenge 3 (2, 4), tension 1 (1, 2), and negative affect 1 (1, 2). In Table 10 and Table 11, we present the scores obtained by *Exogames* and other video games. In Table 12, we present the results of the qualitative information.

## 4. Discussion

We designed and developed *Exogames,* a novel VR exergame for the physical and emotional rehabilitation of landmine victims. During this processes, we identified these relevant aspects to consider for the exergame design:

- The user has concrete thinking: In response to this characteristic, the exergame takes place in a sports field inspired by existing facilities, the athletic track at *Atanasio Girardot* sports complex in Medellín, Colombia. In addition, users perform the usual daily movements while they train and compete.
- Users require a vital dose of motivation, so, in the exergame, users see their own virtual body as complete and healthy, and the trainer is continuously cheering with motivating messages. Additionally, the user listens to the audience’s applause when they meet a goal.
- Competition promotes motivation: The exergame has a scoreboard with users’ ranking that allows each user to compete against others in a time trial, thus avoiding aggression or pressure from other users.
- Motor imagery can support amputee users’ rehabilitation process: The game has a first-person view of a complete body character, including missing limbs, which is the basis for any motor imagery therapy.

About the potential of *Exogames* to support physical rehabilitation, to play this exergame requires the users to perform lower limb joint movements, which are fundamental during the process. According to the perception of a group of health professionals, Figure 7; result of question 1d of Table 2, and 1a, 1b, 1c, and 1g of Table 5; show that 92.3% of these experts agreed or totally agreed that the exercises available in *Exogames* may favor the process of physical rehabilitation of users with lower-limb amputations. Since audiovisual and kinetic stimuli generate sensitive responses [29], an immersive exergame, such as *Exogames*, motivates motor skills, favoring the rehabilitation therapies’ execution. Additionally, shifting the user’s focus on external elements embedded in the rich virtual environment may help the proper execution of exercises, which are frequently painful and tedious. This is why some commercial rehabilitation systems involve video games in their therapy [30]. According to Figure 8, results for questions 1a, 1b, 1c, and 1e of Table 2 and 1d, 1e, 1h, and 1i of Table 5; *Exogames* may promote the welfare of landmine victims. Competition and rewards release dopamine [29], which motivates users to improve their motor skills and promotes therapeutic adherence by establishing achievable objective goals for users. According to the surveys, all of the health professionals considered that the score assigned in this exergame keeps the user’s interest and motivation, and all of them agreed that the experience presented through the exergame was inspiring and engaging. Although VR exergames for rehabilitation exist, to the best of our knowledge, none of the previous research targets the physical and emotional rehabilitation of landmine victims. Considering their complex psychosocial profile, the designed exergame story must avoid retraumatizing them. Some surveyed therapists suggested that emotional therapy for landmine victims is as important as physical therapy, hence, *Exogames* consider the user’s characteristics and provide a quiet environment where the user does not hear outbursts, and danger also does not emerge unexpectedly. It is a competitive virtual environment without a rival or opponent which they may perceive as threatening. Unlike war or terror, sports-related challenges create a sense of safety and order, where serious injuries or death are not present. The exaggerated weightlifting in the virtual exercises and the character’s enhanced cybernetic body empower the user to favor an emotional therapy. Evaluators perceived that such characteristics may make the exergame attractive to users, provide motivation, and promote the right attitude during treatment. As a result, as shown in Figure 8, 92.3% of experts consider that users would find *Exogames* attractive. All of them agreed that a therapy using *Exogames* may psychologically support the users for the physical recovery process, since it is game-based.

*Exogames* supports capturing and processing real-time game data related to exercise execution. These data can improve rehabilitation treatment because the health professional can use it to personalize the process and make corrections; thus enabling obtaining better results, it is even possible that recovery times may be lowered.

According to Table 7, 69.2% of the surveyed health professionals think that *Kina*, including *Exogames*, obtains the necessary user metrics, and 61.5% of them suggest that the user’s performance metrics in the exergame will allow for visualizing the recovery progress, indicating that in-game data collection contributes to improving therapy. Additionally, as shown in Figure 9, 76.9% of experts consider that therapists can extract useful information for the rehabilitation process. In addition, the datasets and the observed results may be valuable for future research related to therapeutic strategies.

To sum up, health professionals reviewed *Exogames* according to the VR game requirements defined in Table 6. As a result, we can see that more than 60% of them consider that it meets the following requirements: All of them report that *Exogames* helps the user to focus on rehabilitation and provides motivation. Moreover, 92.30% expresses that the user feels safe in the virtual world. In addition, 69.23% of them report that the *Exogames* allows them to obtain the user’s metrics. Finally, 61.54% of the health professionals agreed that it can graph the rehabilitation progress.

To complement the initial ad-hoc designed survey, we ran a second assessment, this time using standardized instruments, with five physiotherapists and one person with a lower limb amputation, in terms of usability (SUS), physical activity experience (PACES), and game experience (GEQ). Results show a positive appreciation of the system. Participants indicated a positive effect, a good immersion, good game flow and good competitive feeling during the game experience. The challenge experience was medium. These results agree with the in-game module data. The post-game module, which assessed how users felt after they had stopped playing, demonstrated a good training session with low negative effect, low tension and minor issues in returning to reality. Table 10 and Table 11 show results reported by different articles in the literature [27,28,31] compared to results obtained on this study. For the GEQ questionnaire, *Kina*’s results were superior, showing adequate cohesion of the different aspects of *Exogames* and a positive assessment by users regarding the system. Participants evaluated usability with 69 (66, 79), which is good in the acceptability range. This range indicates whether the evaluated interface of the system is acceptable or not. The average SUS score of other studies is around 68. Finally, PACES results with 110 (108, 112), which means enjoyable. The last assessment suggests that *Exogames* may be used as a virtual reality game for different purposes, including rehabilitation. During this second evaluation, we also asked an open question about qualitative assessment of *Exogames*, see answers in Table 12, regarding the following aspects, which help to support the three contributions presented in this research: a) *Exogames* is highly motivating to exercise, and it may increase the therapy adherence level, and, since the amputee person does not need to support the limb on the floor, *Exogames* could reduce the rehabilitation process duration; b) emotionally, the user may feel that the sensation of disability disappears in this virtual world; and c) about the user interface, the survey suggests that users found the video game easy to use. Additionally, the experts recommended expanding the application field and not limiting it only to landmine victims. They think that *Exogames* has the potential to support therapies for other purposes.

### Limitations

The results presented are mainly based on the perception of health professionals, so therapeutic trials with users should be carried out to corroborate our findings.

*Nukawa* LLRR consider movements only in the sagittal plane. However, the rehabilitation process for victims of antipersonnel landmines requires multiple planes. Therefore, users will need other exercises, besides *Exogames*, for their rehabilitation process.

## 5. Conclusions

*Exogames* is a virtual reality game designed for the physical and emotional rehabilitation of landmine victims. Once integrated with the *Nukawa* lower limb rehabilitation robot, together they constitute the overarching *Kina* system. According to health professionals’ perception, the proposed exergame demonstrates characteristics that may favor physical rehabilitation and the well-being of users with lower-limb amputations. We designed, developed, and evaluated *Exogames* with the help of 18 health professionals and one person with a lower-limb amputation. As a result, we identified the potential contribution of *Exogames* to users’ physical and emotional recovery. *Exogames* may favor the recovery process for landmine victims by building a virtual environment with exercises specially designed for this type of user. Consequently, the highly motivated user may perform the therapeutic exercises in a better way. The surveys distributed to highly qualified and experienced rehabilitation professionals show that they endorse the exergame and perceive that physical and emotional rehabilitation requirements are met. Furthermore, since *Kina* can capture and analyze in-game data, the therapist will be able to adapt the exercises and the therapies to the user’s progress, to improve the recovery process.

To sum up, the main contribution of this paper is that we proposed a VR exergame for the physical and emotional rehabilitation of landmine victims, with the potential to favor the process of physical rehabilitation of users with lower-limb amputations, to promote the welfare of landmine victims, and to support capturing and processing real-time data related to exercise execution during therapy.

Future work involves testing the exergame with users, including monitoring the physical and emotional process, presenting the system’s reports to health professionals, and promoting information-based decisions.

## Figures and Tables

**Table 1:** Questions for the interdisciplinary group in the co-creation exercise.

Q&A Session Questionnaire (Open-Ended Questions)
How could a patient recover from an injury faster and better?
What intensity in movements are needed to have a better recovery?
What color palette is most appropriate for the situation and population?
What sensations and feelings are better?
What kind of significant experience should the subject have?

**Table 2:** Questions about the general perception of the *Kina* system (Likert scale).

Perception Questions (Likert Scale)
1. Taking into account your professional experience, select your concept regarding each statement related to the *Kina* system:
(a) Support the recovery of victims of antipersonnel mines.
(b) Interaction with the system will facilitate adherence to the rehabilitation process for victims of antipersonnel mines.
(c) The game maintains the user’s interest and motivation.
(d) The physical actions of the subject are reflected in the virtual world.
(e) Consider as a positive aspect that the character in the game is healthy and with adequate movements.
(f) If I had a system like *Kina*, I would consider it convenient to use it with my users.
(g) The experience presented was motivating and captivating.
2. Indicate all the conditions that you consider to fulfill the experience that the subject will live with the *Kina* system
(a) It includes familiar settings/objects
(b) It helps to deal with post-traumatic stress
(c) It uses realistic and simplified colors, as the personal environment
(d) It does not overwhelm the user with information or colors
(e) It helps the user to focus on rehabilitation and provides motivation
(f) The user feels safe in the virtual world
(g) It does not generate strong emotional shocks
(h) It does not generate a sense of more disability
(i) It has a scoreboard to motivate the user’s competitiveness
(j) It obtains the user’s metrics
(k) It generates motivation, gratification, independence, and empowerment
(l) It allows to graph the rehab progress statistically
3. Mention any additional observations about the experience

**Table 3:** Questions about intervention areas and graphical interface.

Interventional Areas Questions (Multiple Choice Single Answer Questions)
1. In which stage(s) of transtibial amputation rehabilitation do you think the *Kina* system can contribute the most? (multiple options)
2. *Kina* contributes to the therapeutic objectives framed in the following areas of intervention (multiple options)
Graphical interface questions
3. Please select one answer option for each question (Likert scale)
(a) The graphical interface is intuitive.
(b) The layout of the buttons is visually comfortable
(c) The graphs presented in the interface allow you to extract useful information for the rehabilitation process of victims of antipersonnel mines.
(d) The feedback provided by the graphical interface is sufficient and necessary.
4. What additional information do you think is required to show in the graphical interface?
5. What information presented in the graphical interface do you think can be removed?
6. What additional observations do you have about the graphical interface?

**Table 4:** Survey for health professionals: reports system.

Questions about the Reports System
1. Please select one answer option for each question (Likert scale)
(a) The report presents the information in an easily understandable way
(b) The distribution of information and graphics is adequate in the report
(c) The graphs presented in the report allow us to extract useful information for the rehabilitation process of victims of landmines
2. What additional information do you think is required to be shown in the reporting system?
3. What information presented in the reporting system do you think can be removed?
4. What additional observations do you have about the reporting system?

**Table 5:** *Exogames* questionnarie.

Questions about *Exogames*
1. Please select one answer option for each question (Likert Scale)
(a) According to the modules described, the game allows you to perform flexo-extension movements of the hip, knee, and ankle.
(b) The game is adaptable to the number of sessions defined by the physiotherapist.
(c) The game is adaptable to the exercises selected in each session.
(d) The fact that the therapy is a game psychologically supports the process of physical recovery of the subject.
(e) In my experience as a physical therapist, users would find the game attractive.
(f) The main character looks healthy.
(g) The exercises presented in the game will support the physical rehabilitation process of victims of antipersonnel mines.
(h) I believe that the score assigned in the game maintains the user’s interest and motivation.
(i) The experience presented through the video game was motivating and captivating.
2. What additional observations do you have about the video game *Exogames*?
3. What negative situations that your users have experienced in the rehabilitation process do you think can be reduced or eliminated with the *Kina* system?
4. Additional observations

**Table 6:** List of requirements and perception of compliance.

List of General Requirements	PHP
It helps the user to focus on rehabilitation and provides motivation	100%
The user feels safe in the virtual world	92.3%
It obtains the user’s metrics	69.2%
It allows to graph the rehab progress statistically	61.5%
It generates motivation, gratification, independence, and empowerment	53.8%
It does not generate strong emotional shocks	53.8%
It includes familiar settings/objects	53.8%
It helps to deal with post-traumatic stress	46.2%
It does not generate strong emotional shocks	38.5%
It has a scoreboard to motivate the user’s competitiveness	38.5%
It uses realistic and simplified colors, like the personal environment	30.8%
It does not overwhelm the user with information or colors	23.1%
It does not generate a sense of greater disability	23.1%

**Table 7:** Therapeutic objectives in which *Kina* contributes according to the experts’ perception.

Therapeutic Objectives	
It may allow having user metrics	69.2%
It may allow for visualize the progress in the statistics	61.5%

**Table 8:** Physical rehabilitation therapy stages for transtibial amputation in which physiotherapists consider that *Kina* could be useful.

Rehabilitation Stage	
Prosthetic treatment	38.46%
Pre-prosthetic treatment	30.77%
Postoperative	30.77%
None	0%

**Table 9:** Therapeutic objectives related to intervention areas in which physiotherapist consider *Kina* could be useful.

Intervention Areas	
Arcs of movement	38.46%
Strength	38.46%
Propioception	15.38%
Gait	7.69%
Sensitivity	0%
Pain	0%
Equilibrium	0%
Posture	0%

**Table 10:** *Kina*’s SUS and PACES scores compared to other studies.

Reference	SUS Score	PACES Score	Number of Participants
[24]		123 (18 items)	5
[25]	62	31	153
[26]	68	44	15
*Kina*	69 (66, 79)	110 (108, 112) (18 items)	6

**Table 11:** *Kina*’s GEQ score compared to other studies.

Reference	Flow	Positive Affect	Immersion	Competence	Challenge	Tension	Negative Affect
[27]	3	2.9	2.7	2.6	2.4	0.8	0.4
[28]	1.3	2.3	2	2.2	1.6	0.8	0.3
*Kina*	4 (3, 4)	4 (2, 4)	4 (3, 5)	4 (4, 5)	3 (2, 4)	1 (1, 2)	1 (1, 2)

**Table 12:** Qualitative statements.

About the potential for physical rehabilitation	“It is good to make research for people with these characteristics and motivates to exercise”.
	“Difficulty of adherence to treatment could be decreased or eliminated with the *Kina* system”.
	“Interaction with the virtual environment facilitates adherence issues because it is a novel method and presents challenges to the user”.
	“Have you considered including running?”.
	“The movement given by the equipment could be not only assisted but even resisted”.
	“Not only focus this system on amputee patients. Open the experience to other populations because *Kina* can help the recovery of other populations”.
About the potential to promote welfare of landmine victims	“The sensation of disability could be diminished or eliminated with the *Kina* system”.
	“The person feels safe when is positioned on the system”.
	“With this system, a rehabilitation that would take six months could be done in 20 days or one month because it does not require patient’s foot support”.
	“It can be applied in the future to various pathologies”.
	“I suggest adding a reward system to further motivate the patient”.
	“I suggest creating a competitive strategy that allows motivating the patient to achieve a score according to their needs, it means, to achieve a threshold that in each therapy generates compliance by goals. Ex: biofeedback”.
Interface and reports	“Interaction with the interface is user-friendly and easy to use”.
	“The commands are simple and easy to understand”.

## Data Availability

Not applicable.

## Supplementary Materials

The following supporting information can be downloaded at: https://www.mdpi.com/article/10.3390/s22155602/s1.

## Abbreviations

The following abbreviations are used in this manuscript:

APM	Antipersonnel Landmines
AVM	Antivehicle Mines
AXO	Abandoned Explosive Ordnance
DOF	Degrees of Freedom
ERW	Explosive Remnants of War
GEQ	Game Experience Questionnaire
IED	Improvised Explosive Devices
LLRR	Lower Limb Rehabilitation Robot
PHP	Percentage of Health Professionals
PACES	Physical Activity Enjoyment Scale
Q&A	Question and Answer
SUS	System Usability Scale
UXO	Unexploded Ordnance
VR	Virtual Reality
VRR	Virtual Reality Rehabilitation
